# The relationship between physical exercise and smoking behavior among Chinese residents aged 16 years and older

**DOI:** 10.1038/s41598-023-31511-0

**Published:** 2023-03-20

**Authors:** Yu Tie, Wen Tian, Yiru Chen, Ruiting Wang, Peng Shi, Xiaosu Feng

**Affiliations:** 1 School of Physical Education, Liaoning Normal University, Dalian, China; 2 School of Physical Education, Shanghai University of Sport, Shanghai, China

**Keywords:** Health services, Public health

## Abstract

To explore the relationship between physical exercise and smoking behavior among Chinese residents aged 16 years and older. Analysis based on 29,466 validated cases in the 2018 China Family Panel Studies (CFPS 2018). The chi-square test and Mann–Whitney U test were used for comparative analysis between groups. Logistic regression analysis was used to explore the relationship between physical exercise and smoking behavior. Gender and birth cohort differences in the relationship between physical exercise and smoking behavior were explored based on stratified regression analysis using gender and birth cohort as stratified variables, respectively. Robustness testing based on multiple linear regression analysis using a replacement data approach. There were 8735 cases of smokers among the respondents. After controlling for relevant confounders, there was a significant negative association between physical exercise and smoking behavior among residents [OR 0.718, 95% CI (0.673, 0.765), P < 0.01]. Physical exercise was more significantly associated with smoking behavior among male residents [OR 0.694, 95% CI (0.649, 0.743), P < 0.01], while it was not significantly associated with smoking behavior among female residents [OR 0.901, 95% CI (0.743, 1.093), P > 0.05]. Physical exercise was more significantly associated with smoking behavior in the pre-1948 (OR 0.748), 1959–1968 (OR 0.748), 1969–1978 (OR 0.812), 1989–1998 (OR 0.576) and post-1999 (OR 0.411) birth cohorts, and the association decreased over time and with social change. The results of the robustness test showed that frequency of exercise was significantly and negatively associated with smoking behavior among residents [OR 0.961, 95% CI (0.951, 0.970), P < 0.01]. Physical exercise is negatively associated with smoking behavior among Chinese residents aged 16 years and older, especially among male residents. There is a cohort effect between physical exercise and smoking behavior of the population, that is, the relationship between the two decreases with social change.

## Introduction

Tobacco harm has become one of the most serious public health problems in the world today, and it is the biggest preventable risk factor for human health^1,2^. The occurrence and development of diabetes, stroke, coronary heart disease, malignant tumor, and chronic obstructive pulmonary disease are all associated with tobacco dependence. According to incomplete statistics, smoking causes more than one million deaths from smoking-related diseases in China each year^3^. The tobacco epidemic has brought huge burdens to social and economic development, such as the increase of medical expenses, the decline of productivity and the intensification of poverty. Therefore, tobacco control has become an urgent problem to be solved in the current society.

Physical exercise can play a substitute role for the reward caused by nicotine, and has a positive effect on improving the withdrawal intention, reducing the withdrawal symptoms and reducing tobacco craving^3,4^. Although a large number of clinical experiments^5,6^ have proved that physical exercise can effectively reduce cigarette cravings in tobacco addicts, relieve withdrawal symptoms, and effectively improve the success rate of withdrawal. Currently, the relationship between physical exercise and smoking behavior in China is limited to adolescents^7^ and university students^8^, and there is a lack of evidence on the association between physical exercise and smoking behavior in the Chinese population as a whole. It is therefore difficult to provide sufficient research support for the government to propose a physical exercise assisted smoking cessation policy.

In addition, there may be gender differences and cohort effects in the relationship between physical exercise and smoking behavior among Chinese residents. Because the society is highly tolerant of men, men have a stronger desire to imitate smoking than women, and spend more time unsupervised than women^8^. Some survey studies^9,10^ have also confirmed that men smoke at a much higher rate than women. Similarly, given women’s physical and mental limitations, social image bias and misconceptions about their role in society, numerous studies^11,12^ have shown that women are less likely than men to participate in physical exercise. In addition, the relationship between physical exercise and smoking behavior among the Chinese residents has been complicated by different contexts and social changes. For this reason, we must consider the issue of cohort effects. On the one hand, different birth groups are in different historical periods and there are differences in social systems and economic levels^13^. On the other hand, under the influence of major social changes, different birth cohort groups are at different stages of life and therefore are affected differently^14^. The cohort effect is therefore an important factor in exploring the evolution of the relationship between physical exercise and smoking behavior among Chinese residents from the perspective of social change and life course.

Based on the role of physical exercise in aiding smoking cessation and the lack of evidence yet on the association of physical exercise with smoking behavior in the Chinese residents, this study used the 2018 China Family Panel Study (CFPS 2018) cross-sectional dataset as a sample source for analysis and exploration. Based on the above literature review, this study proposes three research hypotheses. H_1_: Physical exercise is negatively associated with smoking risk in the Chinese residents. H_2_: There were gender differences in the association between physical exercise and smoking risk among Chinese residents, with physical exercise being more significantly associated with smoking risk among male in particular. H_3_: There is a cohort effect for the relationship between physical activity and smoking risk in the Chinese residents. This study establishes preliminary evidence of the association between physical exercise and smoking behavior in the Chinese population, effectively filling a gap in the Chinese population survey and providing research support for the development of exercise assisted smoking cessation policies.

## Methods

### Data sources

The research data in this paper comes from the China Family Panel Studies (CFPS) database (http://www.isss.pku.edu.cn/cfps/download). CFPS is a public dataset implemented by the Institute of Social Science Survey (ISSS) of Peking University. It is a nationwide, large-scale, multidisciplinary social follow-up survey project. Its samples cover 25 provinces/cities/autonomous regions in China. CFPS collects data from three dimensions: individual, family, and community, and adopts various forms of questionnaires such as long, short, pick-up, and telephone interviews, aiming to reflect the development and changes in China’s society, economy, population, health and education^15^. The CFPS did not investigate smoking behavior among children and adolescents aged 15 years and younger, so data from this population were excluded from this study, and 34,743 data were obtained. In addition, 30,202 data were obtained after excluding respondents who did not answer, “not applicable”, “don’t know” and “refused to answer” the smoking and exercise questions. In addition, based on the respondents’ responses as to whether they had ever smoked, there were two groups of people who had ever smoked but were now non-smokers and those who had and were now both non-smokers. Based on the accuracy of the estimation results, the group of ever smokers but current non-smokers was excluded from this study and 29,466 data were obtained. For missing values of control variables, this study used multiple interpolations to fill in the missing values.

### Variable selection

The dependent variable in this study was whether or not they smoked. The CFPS 2018 asked respondents whether they smoked in the past month. In this study, the option “yes” was assigned as 1, the option “no” was assigned as 0. This study used whether or not they participated in physical exercise as the dependent variable. The CFPS 2018 asked respondents how often they participated in physical exercise in the past week. In this study, a frequency of exercise per week of 0 was defined as not participating in physical exercise and was assigned a value of 0. A frequency of exercise per week greater than 0 was defined as participating in physical exercise and was assigned a value of 1. Referring to previous study^16^, the main control variables selected in this study include age, gender, residence, education level, body mass index (BMI), self-perception of health (SPH), socioeconomic status (SES), and life satisfaction. For the examination of cohort effects, this study assigned a value of 1 to the cohort born in 1948 and earlier, a value of 2–6 for every 10 years from 1949 to 1998, and a value of 7 for the cohort born in 1999 and later^17^. In addition, for the regression analysis, this study treated age as a continuous variable. In terms of gender, “male” is assigned a value of 1, and “female” is assigned a value of 0. In terms of residence, “rural” is assigned a value of 0, and “urban” is assigned a value of 1. For education level, this study assigns a value of 1 to “semi-literate/illiterate and primary school”, that is “primary school and below”; 2 to “middle school”; 3 to “high school”; 4 to “college, undergraduate, master and doctor”, that is, “college diploma and above”. For BMI, this study was obtained based on BMI = kg/m^2^. For SPH, SES and life satisfaction, this study assigned a value of 1–5 based on a Likert 5-point scale from low to high, respectively.

### Mathematical statistics

SPSS 25.0 and Stata 12.0 software was used for statistical analysis in this study. In this study, the one-sample Kolmogorov–Smirnov test was used to test the normality of the continuous data. If the data obeyed a normal distribution, the independent samples t-test was used for comparative analysis between groups; if the data did not obey a normal distribution, the Mann–Whitney U test was used for comparative analysis between groups. In addition, this study used the chi-square test for comparative group analysis of categorical variables. In this study, a binary logistic regression analysis was conducted to investigate the relationship between physical exercise and smoking behavior among Chinese residents aged 16 years and above, using “smoking behavior” as the independent variable and “physical exercise” as the dependent variable. In addition, stratified regression analysis was used to explore the heterogeneity of the relationship between physical exercise and smoking behavior among Chinese residents aged 16 years and older, using gender and birth cohort as stratified variables, respectively. Finally, this study examined the relationship between frequency of exercise and smoking behavior among people aged 16 years and older, using “frequency of exercise” as the dependent variable, to test for robustness. The inspection level is defined as α = 0.05.

## Results

### General characteristics of the respondents

Of the 29,466 respondents, 48.8% were males and 51.2% were females; including 50.8% of urban residents and 49.2% of rural residents. Of the 29,446 respondents, 14,338 (about 48.7%) participated in physical exercise, while 8735 (about 29.6%) smoked.

### Smoking prevalence among residents with different characteristics

The differences in smoking prevalence among Chinese residents aged 16 years and older were statistically significant for physical exercise (χ^2^ = 121.892, P = 0.000), gender (χ^2^ = 9457.369, P = 0.000), residence (χ^2^ = 4.768, P = 0.029) and education level (χ^2^ = 70.429, P = 0.000) (Table 1).

**Table 1:** Comparison of smoking prevalence by characteristics of residents.

Characteristics	Options	Number	Number of smokers (%)	χ^2^	P
Physical exercise	Yes	15,128	4052 (26.78%)	121.892	0.000
	No	14,338	4683 (32.66%)		
Gender	Male	15,076	8280 (54.92%)	9457.369	0.000
	Female	14,390	455 (3.16%)		
Residence	Urban	14,487	4209 (29.05%)	4.768	0.029
	Rural	14,979	4526 (30.22%)		
Education level	Primary school and below	12,733	3557 (27.94%)	70.429	0.000
	Middle school	10,202	3228 (31.64%)		
	High school	4546	1463 (32.18%)		
	College diploma and above	1985	487 (24.53%)		

### Age, BMI and subjective perception factors of residents who smoke or not

Age, BMI, SPH, SES and life satisfaction did not follow a normal distribution (P < 0.05), so the Mann–Whitney U test was used for comparative analysis between groups. The differences between smoking and non-smoking residents in age (Z =  − 7.305, P = 0.000), BMI (Z =  − 4.829, P = 0.000), SPH (Z = - − 9.011, P = 0.000), SES (Z =  − 2.173, P = 0.030) and life satisfaction (Z =  − 4.168, P = 0.000) were all statistically significance (Table 2).

**Table 2:** Comparison of age, BMI and subjective perception factors for residents who smoke or not (rank means).

Smoke or not	Number	Age	BMI	SPH	SES	Life satisfaction
Yes	8735	15,291.06	15,102.17	15,392.02	14,576.93	14,432.23
No	20,731	14,498.57	14,578.16	14,456.03	14,799.47	14,860.44
Z		− 7.305	− 4.829	− 9.011	− 2.173	− 4.168
P		0.000	0.000	0.000	0.030	0.000

### The relationship between physical exercise and smoking behavior

Model 1, with no control variables included, shows a negative association between physical exercise and smoking behavior among Chinese residents aged 16 years and older [OR 0.879, 95% CI (0.836, 0.924), P < 0.01]. This negative association between physical exercise and smoking behavior of the residents remained statistically significant after model 2 included control variables [OR 0.718, 95% CI (0.673, 0.765), P < 0.01]. The research hypothesis H1 is supported. In addition, the risk of smoking was higher among residents who were older, male and had higher SPH (P < 0.01); the risk of smoking was lower among residents who were urban, college diploma and above and lower life satisfaction (P < 0.05). Detailed results are shown in Table 3.

**Table 3:** The relationship between physical exercise and smoking behavior among Chinese residents aged 16 years and older.

Independent variable	Model 1		Model 2	
	OR	95%CI	OR	95%CI
Physical exercise				
Yes	0.879**	(0.836, 0.924)	0.718**	(0.673, 0.765)
Age			1.010**	(1.008, 1.012)
Gender				
Male			45.345**	(41.025, 50.119)
Residence				
Urban			0.917*	(0.859, 0.980)
Education level				
Middle school			0.961	(0.893, 1.034)
High school			0.978	(0.890, 1.076)
College diploma and above			0.652**	(0.570, 0.745)
BMI			0.992	(0.983, 1.001)
SPH			1.067**	(1.037, 1.098)
SES			0.980	(0.949, 1.012)
Life satisfaction			0.920**	(0.888, 0.953)

*P < 0.05; **P < 0.01.

### Gender differences in physical exercise and smoking behavior

Model 3 showed a significant negative association between physical exercise and smoking behavior among male residents, after controlling for relevant variables [OR 0.694, 95% CI (0.649, 0.743), P < 0.01]. Model 4 showed a non-significant relationship between physical exercise and smoking behavior among female residents, after controlling for relevant variables [OR 0.901, 95% CI (0.743, 1.093), P > 0.05]. Therefore, there were gender differences in the relationship between physical exercise and smoking behavior among Chinese residents aged 16 years and older, and research hypothesis H_2_ is supported. Detailed results are shown in Table 4.

**Table 4:** Gender differences in the relationship between physical exercise and smoking behavior among Chinese residents aged 16 years and older.

Independent variable	Model 3 (Male: n = 15,076)		Model 4 (Female: n = 14,390)	
	OR	95%CI	OR	95%CI
Physical exercise				
Yes	0.694**	(0.649, 0.743)	0.901	(0.743, 1.093)
Age	1.007**	(1.005, 1.009)	1.035**	(1.029, 1.043)
Residence				
Urban	0.906**	(0.845, 0.972)	0.977	(0.801, 1.192)
Education level				
Middle school	1.021	(0.945, 1.104)	0.727**	(0.572, 0.925)
High school	1.033	(0.935, 1.142)	0.802	(0.580, 1.109)
College diploma and above	0.697**	(0.606, 0.801)	0.411*	(0.207, 0.816)
BMI	0.989*	(0.979, 0.998)	0.998	(0.974, 1.023)
SPH	1.084**	(1.052, 1.118)	0.967	(0.893, 1.047)
SES	0.982	(0.948, 1.106)	0.973	(0.890, 1.062)
Life satisfaction	0.913**	(0.879, 0.948)	0.956	(0.863, 1.059)

*P < 0.05; **P < 0.01.

### Cohort effects in physical exercise and smoking behavior

There was a significant negative association between physical exercise and smoking behavior in the pre-1948 (OR 0.748), 1959–1968 (OR 0.748), 1969–1978 (OR 0.812), 1989–1998 (OR 0.576) and post-1999 (OR 0.411) birth cohorts (P < 0.01). However, physical exercise was not significantly associated with smoking behavior in the 1949–1958 (OR 0.980) and 1979–1988 (OR 0.984) birth cohorts (P > 0.05). In addition, the association between physical exercise and smoking behavior of the residents has gradually decreased over time and with social changes. On this basis, there was a cohort effect of physical exercise and smoking behavior among Chinese residents aged 16 years and older, and research hypothesis H3 was supported. Detailed results are shown in Table 5.

**Table 5:** Birth cohort differences in the relationship between physical exercise and smoking behavior among Chinese residents aged 16 years and older.

Independent variable	Birth cohort (OR)						
	≤ 1948 (n = 2687)	1949–1958 (n = 4944)	1959–1968 (n = 5837)	1969–1978 (n = 5303)	1979–1988 (n = 4902)	1989–1998 (n = 4435)	≥ 1999 (n = 1359)
Physical exercise							
Yes	0.748**	0.980	0.748**	0.812*	0.984	0.576**	0.411**
Gender							
Male	10.884**	30.287**	55.019**	81.536**	108.544**	89.220**	82.135**
Residence							
Urban	0.801*	0.870	1.017	1.082	0.969	0.876	1.183
Education level							
Middle school	0.913	0.869	0.968	0.758**	0.778*	0.661**	0.810
High school	0.561**	0.754*	1.208	0.757*	0.879	0.581**	1.120
College diploma and above	0.514*	0.540**	0.784	0.555**	0.337**	0.478**	1.355
BMI	0.955**	0.925**	0.951**	0.959**	0.998	1.000	1.016
SPH	1.141**	1.139**	1.065*	1.015	1.072	1.093	0.913
SES	1.040**	0.969	1.069	0.969	0.998	0.870**	0.877
Life satisfaction	0.914	0.926	0.914	0.996	0.970	0.927	0.657**

*P < 0.05; **P < 0.01.

### Robustness tests

The reliability of the results is an important basis for the study’s conclusions to be widely accepted. To ensure the robustness of the findings, the study was tested using replacement data. In this study, whether to exercise was replaced with frequency of exercise and a multiple linear regression analysis was constructed for validation. Both model 5 and model 6 showed that frequency of exercise was a protective factor for smoking risk in Chinese residents aged 16 years and older (P < 0.01). It shows that the results of the study are not affected by the parameter setting and statistical methods and have a high degree of external validity. Detailed results are shown in Table 6.

**Table 6:** Results of robustness test.

Independent variable	Model 5		Model 6	
	OR	95%CI	OR	95% CI
Frequency of exercise	0.421**	(0.411, 0.432)	0.961**	(0.951, 0.970)
Age			1.011**	(1.009, 1.013)
Gender				
Male			45.028**	(40.742, 49.764)
Residence				
Urban			0.905**	(0.848, 0.967)
Education level				
Middle school			0.953	(0.885, 1.026)
High school			0.960	(0.873, 1.056)
College diploma and above			0.627**	(0.548, 0.716)
BMI			0.991	(0.983, 1.001)
SPH			1.066**	(1.036, 1.099)
SES			0.977	(0.946, 1.009)
Life satisfaction			0.921**	(0.889, 0.954)

*P < 0.05; **P < 0.01.

## Discussion

The results of this study showed that physical exercise was negatively associated with smoking behavior among Chinese residents aged 16 years and above, which is similar to previous studies^7,8,18^. The neurobiological mechanisms by which physical exercise reduces the likelihood of smoking in the population include the following three aspects. (1) Distraction. According to Tiffany’s selective attention bias theory^19^, smoking behavior is mainly controlled by habit. When smoking behavior is hindered, smokers experience an increase in tobacco craving, accompanied by selective attention to tobacco-related cues, which in turn directs cognitive resources away from the ongoing task to tobacco cues. The mechanism of exercise intervention in smoking cessation is to increase the individual’s cognitive needs and reduce the attention bias caused by tobacco-related cues. (2) Improve cognition. Long-term smoking leads to reduced attention, memory, processing speed, executive function, and atrophy of the hippocampus and prefrontal cortex^14^. However, exercise can alter activation in cognitive-related brain regions, such as the dorsolateral prefrontal cortex and anterior cingulate gyrus, reducing cognitive impairment and improving working memory and inhibition in ex-smokers, thereby reducing tobacco dependence^20^. (3) Reward substitution. Long-term smoking causes the abnormal release of the mesolimbic excitatory neurotransmitter dopamine, leading to dysfunction of the brain’s reward system, which in turn makes it more focused on the positive emotional experience brought about by tobacco. Exercise can change the release of central neurotransmitters such as dopamine, which in turn affects its levels in the central and peripheral blood, resulting in a substitute for the rewarding effect of smoking^21,22^.

There were gender differences in the relationship between physical exercise and smoking behavior among Chinese residents aged 16 years and older, that is, physical exercise was negatively associated with smoking behavior among male residents, but the association with smoking behavior among female residents was not significant. In view of the low social acceptance of female smoking behavior, low smoking imitation desire and risk-taking psychology, and high awareness of health management^11^, the number of female smokers is significantly less than that of male. According to the 2018 Global Adult Tobacco Survey (GATS)^23^, Chinese male current smokers account for 50.5% of the male population, and female current smokers account for 2.1% of the female population. Similarly, this study also proved that gender is an important influencing factor of smoking behavior, and the smoking probability of male is significantly greater than that of female. In addition, female participation in exercise is much lower than that of male due to their physical and psychological limitations, misconceptions about social roles and social stereotypes^11,12^. In conclusion, the lower proportion of female smokers and exercisers may account for the gender differences in this study.

There is a cohort effect for the relationship between physical exercise and smoking behavior among Chinese residents aged 16 years and older, that is, the relationship diminishes over time and social change. The specific health problems of Chinese residents are not only the result of current environmental factors, but also should be traced back to the social and cultural background of their childhood. In an agricultural society, more people and less land is the basic national condition, and planting crops with higher profits is the main means for farmers to improve their livelihoods. In the Ming and Qing Dynasties, tobacco planting, production and operation could obtain higher economic profits, which was an important reason for the rapid spread of tobacco^24^. It soon became an indispensable part of daily life alongside wine and tea and has been handed down to this day^24,25^. After the reform and opening up, with the integration of the tobacco marketing model and the market economy, the disposable income of residents has gradually increased, resulting in a further increase in the proportion of residents who smoke. However, since the beginning of the new century, with the implementation of China’s tobacco control policy, the increase of residents’ health awareness and the improvement of education level, the proportion of young people who smoke in China has shown a downward trend, which may be the reason for the social change that produces the cohort effect.

### The value and significance of this study

This study initially confirmed the negative association between physical exercise and smoking behavior in Chinese residents aged 16 years and older, confirmed that physical exercise is a protective factor against smoking risk in the population, and explained the possible mechanisms by which physical exercise reduces smoking risk at the neurobiological level. This study provides a simple, effective and side-effect-free way for residents to quit smoking, and provides solid data to support the introduction of exercise-assisted smoking cessation policies by the relevant authorities.

### Limitations of this study

From the perspective of sociology, the gender difference of physical exercise on smoking behavior of Chinese residents over 16 years old is only a social phenomenon. The cross-sectional survey can only reflect the correlation between physical exercise and smoking behavior, and cannot establish the causal relationship between them. Therefore, more rigorous epidemiological studies are needed on the effect of physical exercise on smoking behavior in the Chinese residents.

## Conclusion

This study found that physical exercise was negatively associated with smoking behavior among Chinese residents aged 16 years and older, and was a significant protective factor for smoking risk among male residents in particular. Furthermore, this negative association between physical exercise and smoking behavior of the residents decreases over time and with social change. Based on the results of this study, it is recommended that a comprehensive national fitness initiative be implemented to build a social network to support and build an environmental and social support system for exercise participation, to improve motivation and behavior, and to contribute to a “smoke-free society”.

## Supplementary Information

Supplementary Information.

## Data Availability

The datasets used and/or analyzed during the current study available from the corresponding author on reasonable request. All data generated or analyzed during this study are included in this published article and its Supplementary Information files.
